# Micro-Raman spectroscopy study of optically trapped erythrocytes in malaria, dengue and leptospirosis infections

**DOI:** 10.3389/fmed.2022.858776

**Published:** 2022-10-06

**Authors:** Sanu Susan Jacob, Jijo Lukose, Aseefhali Bankapur, N. Mithun, R. Vani Lakshmi, Mahendra Acharya, Pragna Rao, Asha Kamath, Prathap M. Baby, Raghavendra K. Rao, Santhosh Chidangil

**Affiliations:** ^1^Department of Physiology, Kasturba Medical College, Manipal, Manipal Academy of Higher Education, Manipal, India; ^2^Department of Atomic and Molecular Physics, Centre of Excellence for Biophotonics, Manipal Academy of Higher Education, Manipal, India; ^3^Department of Data Science, Prasanna School of Public Health, Manipal Academy of Higher Education, Manipal, India; ^4^Department of Biochemistry, Kasturba Medical College, Manipal, Manipal Academy of Higher Education, Manipal, India; ^5^Department of Physiology, Melaka Manipal Medical College, Manipal Academy of Higher Education, Manipal, India

**Keywords:** red blood cell, micro-Raman spectroscopy, eryptosis, anemia, infectious diseases

## Abstract

Malaria, dengue and leptospirosis are three tropical infectious diseases that present with severe hematological derangement causing significant morbidity and mortality, especially during the seasonal monsoons. During the course of these infectious diseases, circulating red blood cells are imperiled to the direct ill-effects of the infectious pathogen in the body as well as to the pro-inflammatory cytokines generated as a consequence of the infection. RBCs when exposed to such inflammatory and/or pathogenic milieu are susceptible to injuries such as RBC programmed *eryptosis* or RBC programmed *necrosis*. This research aimed to explore the Raman spectra of live red cells that were extracted from patients infected with malaria, dengue, and leptospirosis. Red cells were optically trapped and micro-Raman probed using a 785 nm Diode laser. RBCs from samples of all three diseases displayed Raman signatures that were significantly altered from the normal/healthy. Distinct spectral markers that were common across all the four groups were obtained from various standardized multivariate analytical methods. Following comprehensive examination of multiple studies, we propose these spectral wavenumbers as “*Raman markers of RBC injury*.” Findings in our study display that anemia-triggering infections can inflict variations in the healthy status of red cells, easily identifiable by selectively analyzing specific Raman markers. Additionally, this study also highlights relevant statistical tools that can be utilized to study Raman spectral data from biological samples which could help identify the very significant Raman peaks from the spectral band. This approach of RBC analysis can foster a better understanding of red cell behavior and their alterations exhibited in health and disease.

## Introduction

Malaria, dengue and leptospirosis are tropical infectious diseases that display overlapping clinical and hematological profiles (1). Malaria and dengue require mosquitos as vectors. While malaria is caused by the Plasmodium parasite, infection by the dengue virus (DENV) causes dengue (2). Leptospirosis is a zoonotic disease caused by the bacterial spirochete Leptospira that enters the bloodstream through open wounds from contaminated soil or water bodies (3). Despite the differing causative agents, anemia remains a common hematological aberration observed in the three infections (4–6).

The Plasmodium parasite utilizes red blood cells (RBCs) to complete its life cycle. This can consequently result in hemolysis, which if excessive, can lead to jaundice and anemia (7). Another mechanism that contributes to anemia in malaria is *eryptosis* (8). Eryptosis is a physiological event that is programmed in RBCs, solely meant for the elimination of senescent or abnormal RBCs. This phenomenon, however, is seen to be amplified in many diseases and disorders, as in malaria. RBCs in eryptosis exhibit many structural variations comparable to those seen in apoptosis. Among the many characteristics, some striking features of eryptosis include a decrease in RBC volume leading to its shrinkage, membrane blebbing and vesiculation accompanied by diminution of membrane CD47 expression along with phosphatidylserine (PS) scrambling and exposure. PS-displaying-RBCs are identified by splenic macrophages for phagocytosis. This physiological mechanism is significantly favorable than the alternative RBC death, hemolysis. Hemolysis can be damaging to the body as it releases hemoglobin into circulation, which may adhere to the vasculature, initiate thrombosis and pro-inflammatory activities, and if extensive can even obstruct blood vessels (9). Eryptosis in malaria affects both the parasitized as well as the non-infected RBCs, a process that is activated to impede parasitemia (10).

The pathogenesis of leptospirosis though is mediated mainly through sphingomyelinases. Sphingomyelinases can themselves act as eryptotic stimulators, hydrolyzing membrane sphingomyelin into ceramide (11). Some sphingomyelinases function as hemolysins and are known to cause hemolytic jaundice and anemia, such as in sepsis. Sphingomyelinases are also known to cause deleterious inflammatory effects on a multi-organ/system level (12). Yet another kind of programmed RBC death, distinct from eryptosis is *programmed RBC necrosis*, which is dependent on Fas/FasL signaling, receptor-interacting protein 1 (RIP 1) kinase phosphorylation, and also the mixed-lineage kinase domain-like (MLKL) protein (13). Interestingly, leptospiral infection has been found to activate these signaling mechanisms for hemolysis (14). In contrast to malaria and leptospirosis, dengue has always been associated with platelets and hemorrhagic anemia. However, uncommonly, there have been reports of hemolytic anemia being accompanied by jaundice and reticulocytosis (15, 16). There exists a dearth of knowledge in regards to the effects of dengue infection on RBC physiology. Nevertheless, inducers of eryptosis such as oxidative stress and pro-inflammatory cytokines make strong associations in dengue, as much as they do in malaria and leptospirosis (17).

Jaundice, an unhealthy build-up of bilirubin in the blood, is a frequent finding in malaria, dengue and leptospirosis (18). Hyperbilirubinemia can arise due to hemolysis, liver and/or renal dysfunction. Bilirubin toxicity can itself lead to eryptosis and hemolysis, creating an ambiance for more hyperbilirubinemia (19). The complexity of infection is further intensified with the large legion of cytokines generated during the disease (20, 21). All these factors foster an unhealthy “*eryptotic milieu*” in circulation providing ample probabilities for the RBC to undergo imbalance in its physiology, if not aberrations (22).

RBCs in circulation can display abnormal composition within a pathological environment even though they may appear intact structurally (23). This study intended to investigate the Raman spectral characteristics and chemometrics of RBCs in malaria, dengue and leptospirosis infections. Since the RBC is a very sensitive cell, even a minuscule change in its environment can lead to a drastic variation in its chemical make-up. Therefore, this study necessitated a tool that recorded RBC biochemistry keeping the corpuscles in a physiological medium and that required less-arduous sample-fixation techniques (24). Processing/fixating chemicals could themselves cause cell modifications. Therefore, we employed an in-house assembled micro-Raman spectroscopy system with optical tweezers to record RBC biochemistry *in situ* (25). Raman spectroscopy is an established bio-analytical tool capable of measuring even the slightest molecular change with high sensitivity in real-time (26). The method was standardized in RBCs of hyperbilirubinemic samples wherein the Raman markers that characterized significant variation from the normal were identified and classified using numerous multivariate analytical tests. A detailed description of the technique is available in an earlier article (27).

## Materials and methods

### Ethics statement

This study was approved (IEC 02/2002) by the Institutional Ethics Committee of Kasturba Medical College, Manipal Academy of Higher Education, Manipal and all experimental procedures conformed with the Ethical Committee guidelines. Written informed consent was obtained from all the volunteers. Blood samples of malaria, dengue and leptospirosis patients were collected from the laboratory of Clinical Hematology. The requirement for informed consent was waived concerning the acquisition of patients’ samples.

### Blood sample collection and red blood cell preparation

Four milliliter anticoagulated blood samples were collected from healthy volunteers (*n* = 10), and patients confirmed with malaria (*n* = 10), dengue (*n* = 10) and leptospirosis (*n* = 10) from the laboratory of Clinical Hematology, Kasturba Hospital-Manipal. Blood samples were centrifuged (Labnet Spectrafuge 7M) at 5,000 rpm for 5 min. Following centrifugation, plasma and buffy-coat were aspirated out. 1 μL Hct was pipetted out and suspended in an Eppendorf tube containing 2 mL PBS (Sigma Aldrich, India). 1 μL of RBC suspension in PBS was pipetted onto the sample chamber of the experimental set-up.

A total of 333 raw spectra were recorded from 333 individual RBCs, of which 68 spectra were recorded from the blood samples of healthy volunteers, 97 from that of patients with malaria, 80 from patients with dengue and 88 from patients with leptospirosis.

### Experimental set-up

**FIGURE 1:**
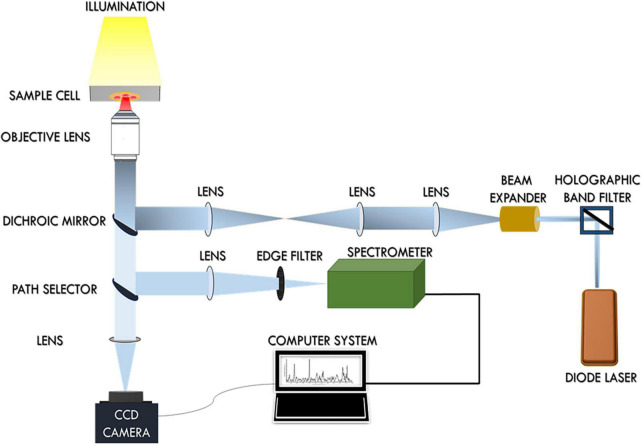
Schematic diagram of the Raman Tweezers set up.

The schematic representation of the single-beam micro-Raman spectrometer combined with optical tweezers has been presented in Figure 1. This tool encompasses an inverted microscope (Nikon Eclipse Ti-U, Japan) coupled with a 785 nm wavelength-emitting diode laser (Starbright Diode Laser, Denmark) and a spectrograph (Horiba Jobin Yvon iHR320). The expanded laser beam overfills the back aperture of a 100 X, 1.3NA oil immersion objective lens (Nikon, Plan Fluor) that creates a well-defined focal spot at the sample plane. RBCs suspended in PBS were optically trapped beneath this focal spot and concurrently Raman-excited. Scattered light from individual RBCs was collected from this same objective and fed into the spectrograph with f-matching optics. The spectrograph was equipped with a grating of dimension 1,200 groves/mm and a liquid nitrogen-cooled charged couple device (Symphony CCD-1,024 × 256-OPEN-1LS) detector of 1,024 × 256 pixels. The removal of Rayleigh scattered light was accomplished by placing an optical edge filter (Razor edge LP02-785RU-25, Semrock, USA) in front of the spectrograph. A detailed account of the experimental arrangement has been previously published (27, 28).

### Data acquisition

The Raman Tweezers setup was employed to optically immobilize and probe RBCs using a 785 nm diode laser. The excitation power of the laser beam was set at ∼10 mW. RBCs were held at focus by adjusting the manual stage. Two accumulations were recorded with an acquisition time of 60 s from each RBC. RBC Spectra were recorded from the fingerprint region (550–1,700 cm^–1^) and further analyzed.

### Data pre-processing

Pre-processing of the accumulated raw RBC spectra was carried out utilizing three software programs, namely, Origin (OriginLab Corp., Northampton, MA) and MATLAB (MATLAB^®^ 7.0).

Pre-processing events comprised omission of spurious noises and cosmic spikes, smoothening of the raw Raman spectra, baseline correction, and vector normalization. The data points that corresponded to cosmic spikes were deleted and further spectral smoothening was performed using the software “Origin.” Spectral baseline correction and normalization were carried out in MATLAB. The second-order polynomial Savitzky-Golay moving-average-technique was employed for spectral smoothening and a two-norm standard-vector normalization technique was employed for normalization (29). The asymmetric Least Squares (AsLS) fitting approach (30) was followed for baseline correction.

### Statistical analysis

Multivariate data analysis was implemented using GRAMS-AI (Grams/AI, PLS Plus IQ) and CRAN R 3.6.1 (^©^ 2019 The R Foundation for Statistical Computing) packages.

#### Partial least-squares discriminant analysis and factor analysis

Partial least-squares discriminant analysis (PLS-DA) of the intensity scores of Raman wavenumbers across the healthy and infectious samples facilitated the extraction of the principal components (PCs). Following this, factor load analysis, which is yet another dimensionality-reduction statistical approach, was implemented on the PCs. Both these tests were carried out using GRAMS spectroscopy software and aided the classification/categorization of the healthy and the three disease samples.

#### MANOVA

The statistically significant difference in the mean value of the Raman intensity scores across the four groups was verified using a non-parametric variant of MANOVA. This analysis was done using CRAN R-3.6.1 with *heplots* (31), *npmv* (32), and *MVN* (33) packages. To enable multiple comparisons, a paired Wilcoxon *post-hoc* test with Bonferonni correction was implemented. The level of significance was set at 0.05 throughout the study.

#### PCA of the significantly different Raman peaks

Subsequently, a PCA was run on the intensity scores of only those wavenumbers that displayed a significant difference in MANOVA. This step was conducted to appreciate the degree of variance between the groups.

#### Factor analysis of principal component

A repeat factor analysis was performed on the PC scores of the healthy and each of the three disease groups. This test displayed a significant distinction between the healthy and each of the disease groups individually.

## Results

### Raman spectra of red blood cells from healthy and disease blood samples

**FIGURE 2:**
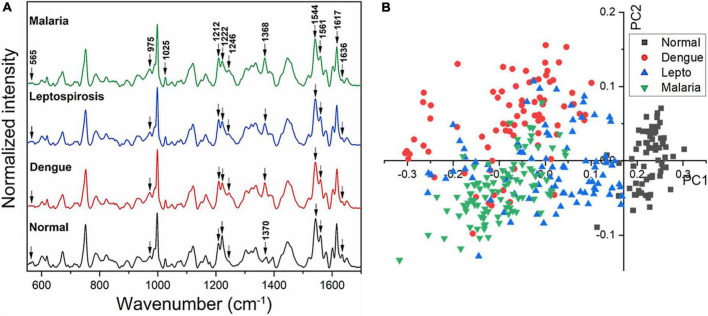
**(A)** Averaged Raman spectra of RBCs from blood samples of healthy volunteers and patients with malaria, dengue and leptospirosis and **(B)** PLS-DA score plot of the first two PCs between RBCs of healthy and the disease samples.

Figure 2A gives the averaged Raman spectra of the healthy and the three disease groups separately. The spectral bands were assigned as was described in earlier studies (34–36) and the details of the vibrational assignments and their variations have been outlined in Table 1. RBCs comprise predominantly of the Hb protein, and hence the spectral records were principally the Raman signatures that emerged from heme. Additionally, contributions from other organic components such as aromatic amino acids, amide bonds and –CH/-CH_2_ side-chains of globular proteins were also obtained that had their origins from the RBC membrane.

**TABLE 1:** Assignments of the significantly different Raman peaks of RBCs from healthy and the three disease samples that were detected through spectral analysis.

Healthy (cm^–1^)	Malaria	Dengue	Leptospirosis	Assignments
565	↓	↓	↓	Fe-O_2_ stretch
(Absent)	*663 (New Peak)*	*663 (New Peak)*	*663 (New Peak)*	p:C-S str (gauche)
752	↓	↓	↓	Pyrrole ring def (out-of-plane), Tryptophan
788	↓	↓	(No change)	Pyrrole ring breathing
975	↑	↑	↑	𝜈(C_*c*_-C_*d*_)
999	↑	↑	↑	Phenylalanine
1,025	↓	↓	(No change)	Phenylalanine
1,049	↑	↑	↑	𝜈(O = O), δ(= C_*b*_H_2_)_*asym*_
1,080	↓	↓	↓	p: C-N str
1,122	↑	↑	(No change)	𝜈(C_β_ -C_1_)_*sym*_
1,165	(No change)	↑	↓	Pyrrole half-ring vibration
1,175	(No change)	↑	↑	Pyrrole half-ring vibration
1,212	↑	↑	↑	methine C_*m*_-H def
1,222	↓	↓	↓	Methine C_*m*_-H def
1,246	↑	↑		p: Amide III (disordered)
1,258	↑	↑	↑	p: Amide III (disordered)
1,264	↑	↑	↑	p: Amide III (disordered)
1,370	1,368 (↑)	1,368 (↑)	1,368 (↑)	Pyrrole half vibration
1,389	↑	↑	↑	Pyrrole half vibration
1,460	↑	↑	↑	δ(= C_*b*_H_2_)_*sym*_, p:δ(CH_2_)
1,524	↓	↓	↓	𝜈(C_β_ -C_β_)
1,544	↓	↓	↓	𝜈(C_β_ -C_β_)
1,561	↓	↓	↓	𝜈(C_β_ -C_β_)
1,602	↓	↓	↓	𝜈(C_α_ - C_*m*_)_*asym*_,𝜈(C_*a*_ = C_*b*_)_*venyl*_
1,617	↓	↓	↓	𝜈(C_α_ -C_*m*_)_*asym*_,𝜈(C_*a*_ = C_*b*_)_*venyl*_
1,636	↓	↓	↓	𝜈(C_α_ -C_*m*_)_*asym*_

p, protein; sym, symmetric; asym, antisymmetric; str, stretch; 𝜈 and δ, in-plane modes; str, stretching, def, deformation. RBCs from healthy volunteers and patients with malaria, dengue and leptospirosis were suspended in PBS and were optically trapped and excited with a 785 nm diode laser. The RBC Raman spectra were captured. The given table lists the frequencies of Raman signals that had displayed increased, decreased and shifted peaks. The assignments of the Raman peaks were carried out based on previous studies.

The averaged spectra between the healthy and the three disease groups revealed numerous variances that encompassed intensity changes and frequency shifts. The common variations exhibited by the RBCs of the disease groups was an increase in intensity of Raman frequencies at 975, 999, 1,049, 1,212, 1,246, 1,258, 1,264, 1,389, and 1,460 cm^–1^ and a decrease in intensity at frequencies 565, 752, 999, 1,080, 1,222, 1,524, 1,544, 1,561, 1,602, 1,617, and 1,636 cm^–1^ when compared to those from RBCs of the healthy group.

### Multivariate partial least-squares discriminant analysis and factor analysis approach

Figure 2B displays the two-dimensional plot of the first two PCs: PC1 and PC2, from the healthy and the three disease groups. This score-plot, generated by Grams IQ, exhibited a clear distinction between the healthy and the three infectious disease groups, indicating that RBCs can be distinguished as “healthy” or “stressed due to a state of disease.” The bi-plot also indicated the existence of certain overlapping PC scores among the disease groups. This implies that despite the three different pathologies, there occurred similarity in the response imparted by the “stressed” RBCs, at least with regards to Raman scattering. Among the three disease groups, the scores of the leptospirosis group exhibited much more variance than those shown by malaria and dengue groups.

We then proceeded to estimate the degree of variance in the Raman intensity scores. In terms of variations in the PCs too substantial differences existed for the classification of RBCs as healthy or “stressed/altered.” The variance captured in this approach, taking into account all the wavenumbers in the spectra, across the four groups, was 52.26%.

**FIGURE 3:**
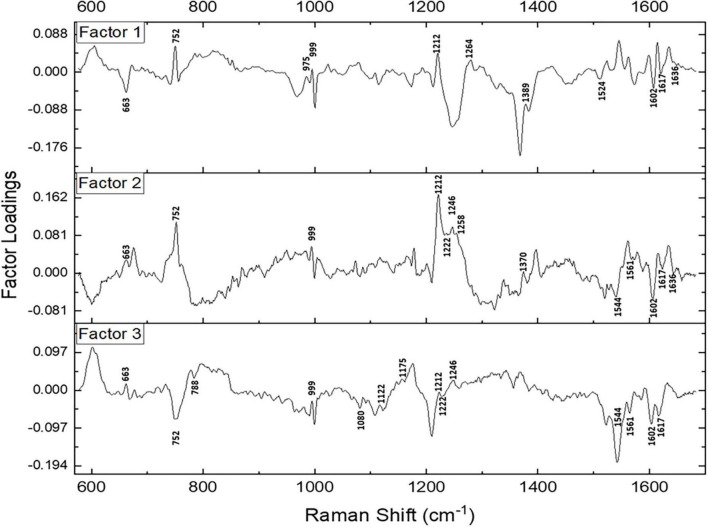
Spectral loadings of the PC factors (Factor 1, Factor 2, and Factor 3) between RBCs of healthy and disease samples.

Subsequently, a factor load analysis of the spectral data was performed. The results (Figure 3) exhibited that all the Raman peaks that showed positive peaks in Factor-1 loading allied to their corresponding peaks of increased intensities in Figure 2A. Similarly, all the Raman signals that showed negative peaks in Factor-1 loading allied with their corresponding peaks with decreased intensities in Figure 2A.

Factors 2 and 3 picked up the subsequent level variations in the spectra, chiefly of Raman peaks at 752, 999, 1,212, 1,222, 1,246, 1,370, 1,389, 1,561, 1,602, 1,617, and 1,636 cm^–1^.

**FIGURE 4:**
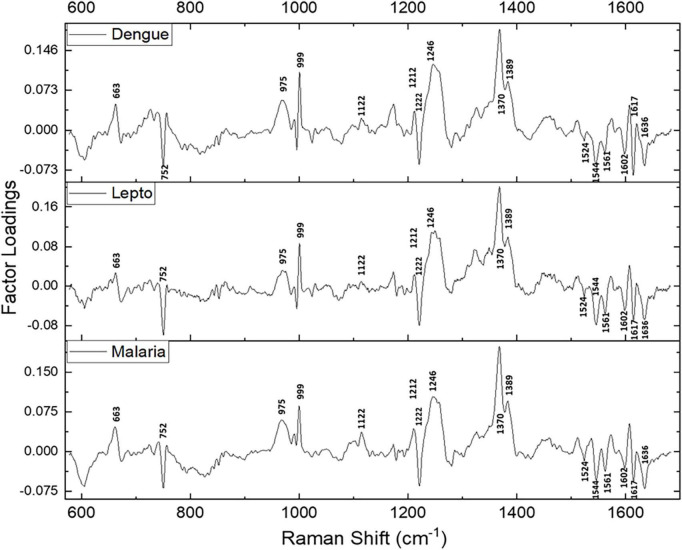
Spectral loadings of the PC1 scores between RBCs of healthy and disease samples.

Since maximum variance had been picked up by PC1, a repeat factor-load analysis was performed for the scores of PC1 across all three infectious disease groups against the healthy group (Figure 4). By performing this repeat factor load analysis, the extent of alterations in the RBC Raman spectra in each disease can be better appreciated.

### Red blood cell Raman markers detected in malaria, dengue and leptospirosis

Spectral analysis, PLS-DA and the factor analysis extracted the Raman frequencies that displayed considerable variations between RBCs from the healthy and the disease populations. The deviant peaks and their assignments have been described below under the four specific band regions, viz., core size/spin-state marker region, pyrrole-ring stretching region, methine C-H deformation region and the low wavenumber region (37). The averaged Raman spectra from the disease groups displayed Raman peaks that had their intensities altered when compared with the healthy group (Figures 2A, 4 and Table 1). The Raman peaks were assigned based on already existing data from previously conducted studies (27, 28, 35) and this paper reports the alteration of these very Raman peaks in malaria, dengue and leptospirosis. The details of the variation in the Raman bands are described and elaborated below.

#### Core size or spin state marker band: 1,500–1,650 cm^–1^

This marker band encompasses the Raman signatures that disseminate from the C-C bonds in the porphyrin ring of heme and is dependent on the spin-state of atomic iron (38). Raman peaks that signify the “oxy” state of Hb at 1,524, 1,544, 1,561, 1,602, 1,617, and 1,636 cm^–1^ exhibited a decrease in their intensities in all three disease spectra as compared to their corresponding peaks in the healthy spectra. This inferred a decline in the “oxy” configuration of heme (21) comprising Hb with a reduced amount of bound-oxygen in disease. Raman peak 1,636 cm^–1^ characterizes oxygen concentration, a decrease of which implied a fall in the oxyconfiguration of heme (39).

#### Pyrrole-ring stretching band: 1,300–1,400 cm^–1^

The importance of this spectral zone is that it includes the heme-aggregation bands and also conveys information relating to the “oxidation” state of iron in heme. In all three disease groups there appeared to be a notable increase in the intensity of the heme-aggregation Raman peak at 1,368 cm^–1^ accompanied by an enhanced pyrrole deformation peak at 1,389 cm^–1^. This occurrence indicated the production of heme-aggregates in RBCs (40).

#### Methine C-H deformation band: 1,200–1,300 cm^–1^

This zone of the Raman spectrum is attributed to the methine C-H bonds within heme. These bonds are sensitive to alterations in Hb conformations and are influenced by the binding of oxygen with heme iron. Their closeness with the protein subunits is inferred to be the basis of their sensitivity (35). Raman peaks that displayed enhanced intensity in this spectral region were wavenumbers at 1,212, 1,246, and 1,264 cm^–1^. Alteration of 1,246 cm^–1^ is indicative of heme aggregation (41). This was accompanied by a fall in intensity at the peak of 1,222 cm^–1^ suggesting changes in the methine C-H deformation (35, 36).

#### Low wavenumber band: 600–1,200 cm^–1^

Pyrrole-breathing and pyrrole-deformation features of RBCs can be studied from this Raman region (42). This portion of the Raman spectrum also depicts membrane stability (43, 44). There appeared a significant increase in the intensity of the Raman peak at 975 cm^–1^ in all three disease groups. A new peak surfaced at 663 cm^–1^ that shouldered the 675 cm^–1^ pyrrole-deformation peak across the disease populations as well. Both these events are indicative of heme-aggregation in consequence of protein denaturation (40, 41). The porphyrin breathing mode peak at 752 cm^–1^, signifying Hb integrity (45), exhibited a significantly reduced intensity in the disease groups. The deoxy Raman marker peak at 788 cm^–1^ displayed a substantial decrease in intensity in all three disease groups (28). The peaks originating from phenylalanine at 999 and 1,025 cm^–1^ Raman peaks displayed a deviation in their strengths when compared to the healthy spectrum. This implied Hb breakdown (38, 46) and disruption of the RBC membrane (43). Altered heights of peaks 1,122 and 1,080 cm^–1^ in the disease groups that emanate from the RBC membrane signified distorted membrane deformability (41), whereas altered intensities at 1,165 and 1,175 cm^–1^ characterize deformation in the pyrrole ring (47).

### PCA of the significantly different wavenumber bands

On the whole, there were 26 Raman bands (Table 1) detected by the spectral analysis approach that presented a significant difference between the healthy and disease samples. A repeat PCA, in CRAN R-3.6.1, of the intensity-scores of just these significantly different Raman peaks captured a better variance of 68.13% for the first three PCs, much higher than that obtained (52.26%) when the whole molecular fingerprint region of the RBC spectra was considered.

**FIGURE 5:**
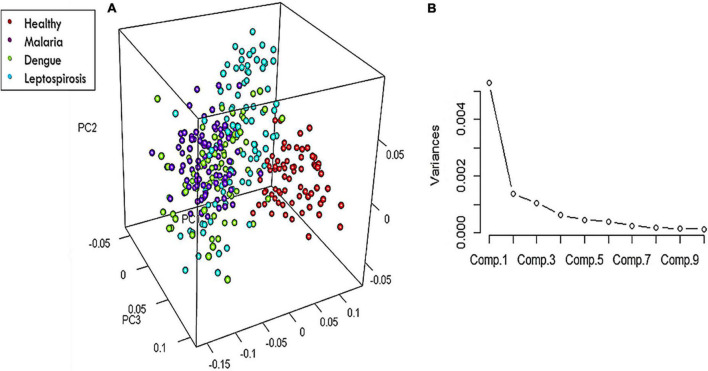
**(A)** 3-Dimensional score-plot for the healthy and the three disease samples and **(B)** % Variance of the PCs.

The 3-dimensional score-plot of the first three PCs displayed in Figure 5A exhibited a clear classification of the four groups. Figure 5B displays the scree-plot of the first ten PCs which exhibited that among the first ten PCs, the first PC captured maximum variance (46.62%) from the data procured from the four groups.

### Non-parametric MANOVA approach to Raman spectral markers

A non-parametric variant of MANOVA (CRAN R 3.6.1) was used to investigate the statistically significant difference in the mean intensity scores across the four groups for only those Raman bands that were identified as significantly different in the spectral analysis. Toward this, the McKeon approx. for Lawley Hotelling Test was considered. Results indicated a significant difference (*p*-value < 0.00001) between the average values of the intensity scores across the healthy and all three disease samples. The Lawley-Hotelling trace test in MANOVA was employed to examine the discriminating aspects of the groups that encompass large datasets.

Subsequently, a pairwise Wilcoxon test with Bonferonni corrections was utilized to facilitate *post hoc* comparisons. This helped identify those Raman peaks that attributed to significant differences in the intensity scores across the four populations. The *p*-values that were obtained have been presented in Table 2 for the following pair of groups: Group 1 (healthy) vs. Group 2 (dengue), Group 1 (healthy) vs. Group 3 (leptospirosis), Group 1 (healthy) vs. Group 4 (malaria), Group 2 (dengue) vs. Group 3 (leptospirosis), Group 2 (dengue) vs. Group 4 (malaria), Group 3 (leptospirosis) vs. Group 4 (malaria).

**TABLE 2:** Level of significance of the significantly different Raman peaks derived from pair-wise Wilcoxon *post-hoc* test between RBCs of healthy and the disease samples.

	*P-value*					
Raman Peak (cm^–1^)	Group 1 vs. Group 2	Group 1 vs. Group 3	Group 1 vs. Group 4	Group 2 vs. Group 3	Group 2 vs. Group 4	Group 3 vs. Group 4
***565***	<0.00001	0.09	<0.00001	0.00019	1.00	<0.00001
***663***	<0.00001	0.00012	<0.00001	0.00013	1.00	0.002
***752***	<0.00001	<0.00001	<0.00001	<0.00001	<0.00001	0.69
***788***	0.00026	1.00	<0.00001	0.00146	1.00	<0.00001
***975***	<0.00001	0.001	<0.00001	<0.00001	0.094	<0.00001
***999***	<0.00001	<0.00001	<0.00001	0.00013	0.457	0.00094
***1,025***	<0.00001	0.32	0.10	0.010	0.011	1.00
***1,049***	0.04	0.91	1.00	1.00	1.00	1.00
***1,080***	<0.00001	0.00269	0.00073	1.00	0.785	1.00
***1,122***	0.10	1.00	0.25	0.0025	1.00	0.01
***1,165***	0.0230	1.00	1.00	0.0003	0.10	0.30
***1,175***	0.0190	0.3832	1.00	0.0013	0.10	0.56
***1,212***	<0.00001	0.3726	<0.00001	<0.00001	0.144	0.0002
***1,222***	<0.00001	<0.00001	<0.00001	0.90	0.74	1.00
***1,246***	<0.00001	<0.00001	<0.00001	<0.00001	1.00	<0.00001
***1,258***	<0.00001	<0.00001	<0.00001	0.00038	1.00	<0.00001
***1,264***	<0.00001	<0.00001	<0.00001	<0.00001	1.00	<0.00001
***1,370***	<0.00001	<0.00001	<0.00001	0.036	0.019	<0.00001
***1,389***	<0.00001	<0.00001	<0.00001	0.022	0.799	<0.00001
***1,460***	<0.00001	0.0012	<0.00001	0.0224	1.00	0.039
***1,524***	<0.00001	<0.00001	<0.00001	<0.00001	<0.00001	<0.00001
***1,544***	<0.00001	<0.00001	<0.00001	<0.00001	<0.00001	<0.00001
***1,561***	<0.00001	<0.00001	<0.00001	<0.00001	<0.00001	<0.00001
***1,602***	<0.00001	<0.00001	<0.00001	<0.00001	<0.00001	<0.00001
***1,617***	0.39	0.00072	<0.00001	0.32	0.0009	<0.00001
***1,636***	<0.00001	<0.00001	<0.00001	1.00	0.029	0.014

Group 1: Healthy, Group 2: Dengue, Group 3: Leptospirosis, Group 4: Malaria. The underlined *p*-values represent those that did not exhibit a significant difference (*p* > 0.05) in the *post hoc* analysis between the intensity scores in that pair.

#### Group 1 (healthy) vs. group 2 (dengue)

Excluding the Raman peak at 1,617 cm^–1^ (*p* = 0.39) that characterizes the “oxy” state of Hb, the intensity scores of all the other Raman bands detected by spectral analysis exhibited a highly significant difference between the healthy and dengue samples.

#### Group 1 (healthy) vs. group 3 (leptospirosis)

Between the healthy and leptospirosis groups, excluding the following Raman peaks: oxygenation status marker at 565 cm^–1^ (*p* = 0.09), Hb integrity conferring marker at 788 cm^–1^ (*p* = 1.00), phenylalanine and amide emanating peaks at 1,025 cm^–1^ (*p* = 0.32) and 1,049 cm^–1^ (*p* = 0.91), respectively, RBC membrane peak at 1,122 cm^–1^ (*p* = 1.00) and the pyrrole deformation peaks at 1,165 cm^–1^ (*p* = 1.00) and 1,175 cm^–1^ (*p* = 0.38), all other Raman peaks exhibited significant differences across the samples.

#### Group 1 (healthy) vs. group 4 (malaria)

Amongst the healthy and malaria samples, except the Raman peaks at 1,025 cm^–1^ (*p* = 0.10) from phenylalanine, 1,049 cm^–1^ (*p* = 1.00) from amide, and the pyrrole deformation peaks at 1,165 cm^–1^ (*p* = 1.00) as well as 1,175 cm^–1^ (*p* = 1.00), all other Raman peaks appeared significantly different.

On the whole, among the three diseases, RBCs from the dengue samples exhibited significant variances from the healthy in all Raman markers, excluding one, as compared to those from malaria and leptospirosis samples. For completeness, a pairwise comparison of the Raman markers between the disease groups was also conducted which generated the following results:

#### Group 2 (dengue) vs. group 3 (leptospirosis)

A pair-wise comparison between the dengue and leptospirosis populations exhibited substantial distinction between the two groups, excluding Raman peaks at 1,049 cm^–1^ (*p* = 1.00) which emanated from amide, 1,080 cm^–1^ (*p* = 1,00) a membrane deformability marker, 1,222 cm^–1^ (*p* = 0.90) from the methine C-H deformation band and 1,636 cm^–1^ (*p* = 1.00) an “oxygenation” status Raman marker.

#### Group 3 (leptospirosis) vs. group 4 (malaria)

Between malaria and leptospirosis groups too, excluding Raman peaks at 752 cm^–1^ (*p* = 0.69) a porphyrin breathing mode peak, phenylalanine emanating peak at 1,025 cm^–1^ (*p* = 1.00), amide emanating peak at 1,049 cm^–1^ (*p* = 1.00), the membrane deformability marker at 1,080 cm^–1^ (*p* = 1.00), pyrrole deformation peaks at 1,165 cm^–1^ (*p* = 0.30) as well as at 1,175 cm^–1^ (*p* = 0.56) and the methine C-H deformation band at 1,222 cm^–1^ (*p* = 1.00), there was significant distinction.

#### Group 2 (dengue) vs. group 4 (malaria)

Between dengue and malaria groups, there appeared many Raman peaks that exhibited lesser variance such as the oxygenation status markers at 565 cm^–1^ (*p* = 1.00), heme-aggregation denoting peak at 663 cm^–1^ (*p* = 1.00), the deoxy Raman marker peak at 788 cm^–1^ (*p* = 1.00), heme aggregation peak at 975 cm^–1^ (*p* = 0.90) and phenylalanine emanating peak at 999 cm^–1^ (*p* = 0.46), RBC membrane peak at 1,122 cm^–1^ (*p* = 0.79), pyrrole deformation peaks at 1,165 cm^–1^ (*p* = 1.00) as well as 1,175 cm^–1^ (*p* = 0.10), the methine C-H deformation bands at 1,212 cm^–1^ (*p* = 0.14) and 1,222 cm^–1^ (*p* = 0.74), 1,246 cm^–1^ (*p* = 1.00), 1,258 cm^–1^ (*p* = 1.00), and 1,264 cm^–1^ (*p* = 1.00) and the pyrrole deformation peaks at 1,389 cm^–1^ (*p* = 0.80) and 1,460 cm^–1^ (*p* = 1.00).

As seen above, it is clear that pairwise comparisons between disease groups displayed those Raman peaks that showed significant deviation from the normal/healthy, but not from each other. This is an indication that these deviations could be generic and not specific to the disease. On the other hand, those Raman peaks that displayed significant distinction between the diseases in the pair though could be an exclusive or elite feature of the particular disease on RBCs.

## Discussion

To summarize the key findings of this study, in malaria, dengue and leptospirosis, variations were observed in the intensities of Raman peaks that represented the “oxygenation status” of Hb. It was observed that these Raman signatures primarily exhibited a “*deoxy*” configuration configuration rather than an “oxy” configuration, as compared to RBCs in the healthy samples. There also occurred a decline in the frequencies signifying “oxygen saturation” in the disease samples (28). Another characteristic feature observed in RBCs from diseased samples is that they showed a predominance of those Raman markers indicative of heme-aggregation (39). Results also showed a significant drop in peak intensities that depicted Hb stability in conjunction with altered Raman peaks that portrayed RBC membrane stability (38, 43, 46).

It is vital that Hb be biochemically conducive enough for oxygenation, as this helps RBCs to accomplish its oxygen-transporting-role (48). Hb, having taken on more of a “deoxy” configuration, with diminution in its oxygen saturation status and the presence of heme aggregation, are all suggestive of a disturbance in its “healthy” state in disease, possibly reflecting in its physiology as well. Taking into consideration all the Raman variations across the healthy and disease groups, it is difficult not to conclude that infection does affect RBC biochemistry, either directly or indirectly. Studies have shown that RBCs exhibit programmed death processes such as eryptosis and/or erythrocyte programmed necrosis in malaria and in diseases that generate excessive pro-inflammatory products (49). The results of this study further support this datum. However, in the future, it is to be investigated if distinctive RBC Raman signatures exist with respect to specific infections. Another potential limitation of this study is that the excitation power of the laser beam was set approximately at 10 mW. It is possible that this excitation power, even if stated as non-injurious in many studies (50, 51), can pose as a source of stress to the RBCs, if not damage. This could possibly add on more strain to the already stressed and sensitive RBCs in disease. In this regard, in a future study we intend to utilize laser beams of smaller excitation powers to further investigate RBC Raman signatures in disease.

When a repeat multivariate analysis was performed, considering the picked out Raman peaks that exhibited significant difference among the four populations, a better variance was captured between the groups. This signified that the selective analysis of merely these Raman peaks was adequate to categorize RBCs as healthy or stressed. These identified Raman peaks were the same ones that appeared to be of significance in the micro-Raman analysis of RBCs in jaundice samples, in an earlier study (27). Former reports, pertaining to RBC Raman scattering analysis in eryptosis, also point out changes in these very peaks (34, 52). With the analysis we present in this report and the standardized study we had conducted earlier (27), we hypothesize these peaks as “*Raman markers of RBC injury*.” Therefore, to identify RBCs under stress/injury, consideration of these Raman peaks would suffice than running chemometrics over all of the Raman peaks in the 550–1,700 cm^–1^ Raman region. That being said, it is essential to conduct and validate the technique on other pathologies and *in vitro* experiments of eryptosis, to establish this statement conclusively.

## Conclusion

Spectral analysis and the various multivariate analytical methods employed, as was standardized in our earlier study (23) disclosed that RBCs in malaria, dengue and leptospirosis infections endured biomolecular deviances from the normal. Intensity scores derived from the RBC Raman peaks were run through numerous multivariate analytical approaches to confirm the same. As a preliminary study, the systematic use of multivariate tools and the results obtained validates this method to investigate RBC Raman signatures. Deciphering the chemical nature of the RBCs in their exact/precise state is a demanding task. Nevertheless, a tool such as Raman spectroscopy can achieve this, holding great promise to be a potential diagnostic tool in the medical domain.

## Data availability statement

The datasets generated and analyzed during this study are available from the corresponding author on reasonable request.

## Ethics statement

The studies involving human participants were reviewed and approved by the Kasturba Medical College and Kasturba Hospital Institutional Ethics Committee, Registration No. ECR/146/Inst/KA/2013/RR-16. Written informed consent was obtained from all the volunteers. Blood samples of malaria, dengue and leptospirosis patients were collected from the laboratory of Clinical Hematology. The requirement for informed consent was waived concerning the acquisition of patients’ samples.

## Author contributions

SJ, PR, and SC conceived and designed the study. SJ, JL, and NM performed the experiments, interpreted the results, and wrote the manuscript. AB performed the multivariate analysis using GRAMS-IQ and the interpretation of the results. MA contributed to performing the experiments. AK and RV contributed to the statistical analysis and interpretation of results and wrote the manuscript. RR and PB contributed to the conception and design of the project and the interpretation of results. All authors contributed to the article and approved the submitted version.
